# Survey of Deoxynivalenol Contamination in Agricultural Products in the Chinese Market Using An ELISA Kit

**DOI:** 10.3390/toxins11010006

**Published:** 2018-12-24

**Authors:** Ming Li, Mingna Sun, Xia Hong, Jinsheng Duan, Daolin Du

**Affiliations:** 1 Institute of Environmental Health and Ecological Security, School of the Environment and Safety Engineering, Jiangsu University, Xuefu Road 301, Zhenjiang 212013, China; liming@ujs.edu.cn (M.L.); hongxiaujs@hotmail.com (X.H.); 2 Institute of Plant Protection and Agro-Product Safety, Anhui Academy of Agricultural Sciences, Key Laboratory of Agro-Product Safety Risk Evaluation (Hefei), Ministry of Agriculture, South Road of agricultural science 40, Hefei 230031, China; sunmingna@aaas.org.cn (M.S.); duanjinsheng@aaas.org.cn (J.D.)

**Keywords:** deoxynivalenol, contamination, survey, ELISA kit, agricultural product

## Abstract

A total of 328 agricultural product samples highly suspected to be contaminated, from flour companies, feed companies, and livestock farms throughout China, were surveyed for deoxynivalenol (DON) contamination using a self-assembly enzyme-linked immunosorbent assay (ELISA) kit. An ELISA kit for DON was developed with a 4.9 ng mL^−1^ limit of detection (LOD) in working buffer and a 200 ng g^−1^ LOD in authentic samples. The DON contamination detection rate was 88.7%, concentrations ranged from 200.9 to 6480.6 ng g^−1^, and the highest DON contamination was found in distillers’ dried grains with solubles with an average of 3204.5 ng g^−1^. Wheat bran and wheat were found to be the most commonly contaminated samples, and the corn meal samples had the lowest average DON level. This ELISA kit is a powerful alternative method for the rapid, sensitive, specific, accurate, and high-throughput determination of DON and can meet the maximum requirement levels. This survey suggests that DON contamination in the Chinese market is serious, and the contamination risk deserves attention. Essential preventive measures should be implemented to ensure food safety and human health.

## 1. Introduction

Deoxynivalenol (DON), a highly toxic secondary metabolite mainly produced by *Fusarium graminearum* and *Fusarium culmorum*, may threaten the health of humans and animals, as it acts as an antifeedant and demonstrates immunotoxicity, organ toxicity, inhibition of protein synthesis, and teratogenicity [1,2,3]. DON contaminates wheat, barley, corn, and other cereal crops and food products worldwide [4,5]. Its co-toxic effect with other mycotoxins such as aflatoxin cannot be ignored [6]. Given its serious toxic effects, the European Commission (EC) published a tolerable daily intake for DON of 1 μg kg^−1^ of body weight per day [7], and the European Union (EU) set the DON maximum levels (MLs) at 1250 ng g^−1^ and 750 ng g^−1^ in unprocessed cereals and food, respectively [8]. In China, the ML of DON in corn, wheat, and their products was regulated at 1000 ng g^−1^ [9].

To monitor and control the DON contamination in cereals and other agricultural products, various analytical methods for DON are used. These mainly include thin-layer chromatography (TLC) [10], gas chromatography (GC) [11], high-performance liquid chromatography (HPLC) [12], GC tandem mass spectrometry (GC-MS) [13], and HPLC-MS [14]. These mentioned methods are standardized with high precision and sensitivity, but require technical expertise, and they are expensive, time-consuming, and unsuitable for screening of DON for large numbers of samples [15]. Immunoassays are simple, rapid, and cost-effective, with adequate sensitivity and high selectivity [16,17,18,19,20]. Series immunoassays, such as conventional colorimetric enzyme-linked immunosorbent assay (ELISA) [21,22,23], chemiluminescence enzyme immunoassay (CLEIA) [24], fluorescence polarization immunoassay (FPIA) [25], time-resolved fluoroimmunoassay (TRFIA) [26,27], and the gold immunochromatographic assay (GICA) [28], were developed to detect DON. Later, the surface plasmon resonance (SPR) immunoassay [29,30], silver staining GICA [31], nanobody-based ELISA [32], and immunosensor [33,34] for DON were introduced.

As a fundamental immunoassay, ELISA has been widely developed for detecting DON, given its simplicity, specificity, low cost, and ability to perform high-throughput screening [35,36,37]. Furthermore, rapid-detection technologies such as ELISA have received more attention; it is an alternative or complementary detection means that has been accepted by consumers and quarantine officers [38,39,40]. Due to the prevalence of mycotoxin contamination and the large number of samples that need to be analyzed, ELISA kits have been considered a suitable screening tool for the determination of mycotoxins, and their development and application has grown rapidly in recent years.

The goal of this study was to survey DON contamination in agricultural products in the Chinese market using a sensitive ELISA kit. A DON ELISA kit was assembled and debugged in our laboratory. The developed ELISA kit showed to be sensitive, specific, quantitative, and capable of screening a large number of DON-contaminated samples. The developed DON ELISA kit was applied to the analysis of 328 agricultural product samples that were highly suspected to be contaminated, including wheat flour, distillers’ dried grains with solubles (DDGS), feed, corn, wheat bran, wheat, and corn meal samples. The contamination levels of DON in these selected samples were evaluated. The accuracy of the ELISA kit was validated in comparison with HPLC.

## 2. Results and Discussion

### 2.1. Sensitivity

**Figure 1:**
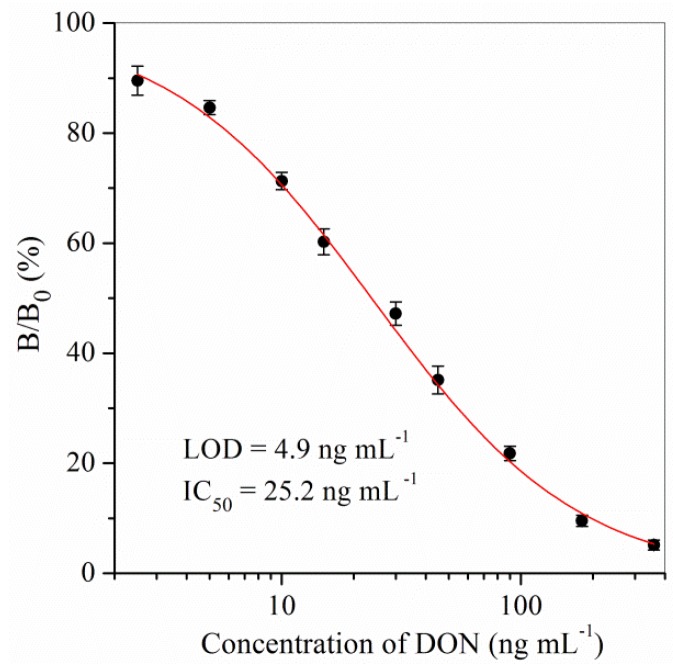
ELISA kit calibration curve for DON. ELISA, enzyme-linked immunosorbent assay; DON, deoxynivalenol; B/B_0_ (%), the ratio value of absorbance with DON and in the absence of DON; LOD, limit of detection; IC_50_, half-maximal inhibition concentration.

The standard curve of the ELISA kit for DON was constructed under optimum conditions (Figure 1). The proposed ELISA kit for DON had a limit of detection (LOD, IC_15_) of 4.9 ng mL^−1^, a half-maximal inhibition concentration (IC_50_) of 25.2 ng mL^−1^, and a linear range (IC_15_–IC_85_) of 4.9–128.9 ng mL^−1^.

In previous studies, DON contamination was determined using TLC methods, and the LODs were more than 40 ng g^−1^ [10]. The GC method has been used to detect DON in corn, and the LOD was 10 ng g^−1^ [11]. The LOD of the HPLC method equipped with immunoaffinity purification for DON in wheat reached 10 ng g^−1^ [12]. The HPLC-MS method also has been used to detect DON, and the LOD was 25 μg kg^−1^ [14]. The key properties of the referenced immunoassays for DON are listed in Table 1. The sensitivity of the proposed ELISA kit for DON is much higher than those of most of the above instrumental methods and referenced ELISAs [2,21,35], but is not as good as those of some novel ultrasensitive methods [24,27,34]. On the whole, the sensitivity of the proposed ELISA kit is sufficient to effectively detect DON.

### 2.2. Specificity

As shown in Table 2, the cross-reactivity (CR) of the ELISA kit toward related mycotoxins was negligible (5.7% for 3-acetyldeoxynivalenol and under 0.5% for others), indicating high specificity. Therefore, the negligible CRs between DON and its related mycotoxins enabled the development of an ELISA kit for the specific determination of DON.

### 2.3. Accuracy and Precision

In the process of immunoassays for authentic samples, matrix effects are the most common challenges. The dilution method was used to minimize the matrix effects in this study. With the increase in dilution, the matrix effects on the sensitivity were reduced. When the matrixes were 1:40 dilutions, the matrix effects of these selected samples on the sensitivity of the ELISA kit could be ignored. A 40-fold dilution was used for subsequent DON determination in agricultural product samples. Thus, the LOD of the ELISA kit was 200 ng g^−1^ in authentic samples. The official MLs of DON have been previously published [7,8,9]. The LOD of the ELISA kit was much lower than the lowest ML level of 750 ng g^−1^ in food [8], which indicated that the sensitivity of ELISA kit can meet the requirement for the analysis of DON contamination.

As demonstrated in Table 3, the recoveries of DON by the ELISA kit in spiked samples ranged from 76.3 to 114.5% with the relative standard deviations (RSDs) ranging from 3.9 to 13.2%. These results suggested that the accuracy and precision of the proposed ELISA kit was satisfactory for the survey of DON contamination in the selected samples.

### 2.4. Determination and Evaluation of Authentic Samples

A total of 328 samples highly suspected of being contaminated from flour companies, feed companies, and livestock farms were collected. These samples were categorized as flour, feedstuffs, and foodstuffs products. Flour included wheat flour and corn meal. Feedstuffs included DDGS, feed, and wheat bran. Foodstuffs included corn and wheat. As shown in Table 4, 291 samples (88.7% of 328 samples) were found to contain DON, having concentrations ranging from 200.9 to 6480.6 ng g^−1^; 37 samples (11.3% of 328 samples) had DON concentrations of less than 200 ng g^−1^; 146 samples (44.5% of 328 samples) had DON contamination levels ranging from 200 to 1000 ng g^−1^; 102 samples (31.1% of 328 samples) had DON contamination levels ranging from 1000 to 3000 ng g^−1^; and 43 samples (13.1% of 328 samples) had DON concentrations above 3000 ng g^−1^.

DON contamination was found in 91.8% of 135 wheat flour samples, 94.1% of 17 DDGS samples, 83.9% of 56 feed samples, 87.2% of 47 corn samples, and 63.0% of 27 corn meal samples. A total of 15 wheat bran samples and 31 wheat samples had detected DON contaminations above 200 ng g^−1^, which were the highest detection rates. The corn meal samples had the lowest average DON concentration, and 37% of 27 corn meal samples had no detected DON, which was the lowest DON detection rate of 63%. The highest DON concentrations were found in DDGS samples, ranging from 1201.3 to 6480.6 ng g^−1^, with an average of 3204.5 ng g^−1^. Wheat flour samples and wheat bran samples were heavily contaminated with DON. These observations indicated that DON contamination is a commonly occurring problem in agricultural products in the Chinese market that should be strictly monitored, and effective controls should be implemented to ensure food safety and human health.

### 2.5. Correlation of ELISA Kit and HPLC

**Figure 2:**
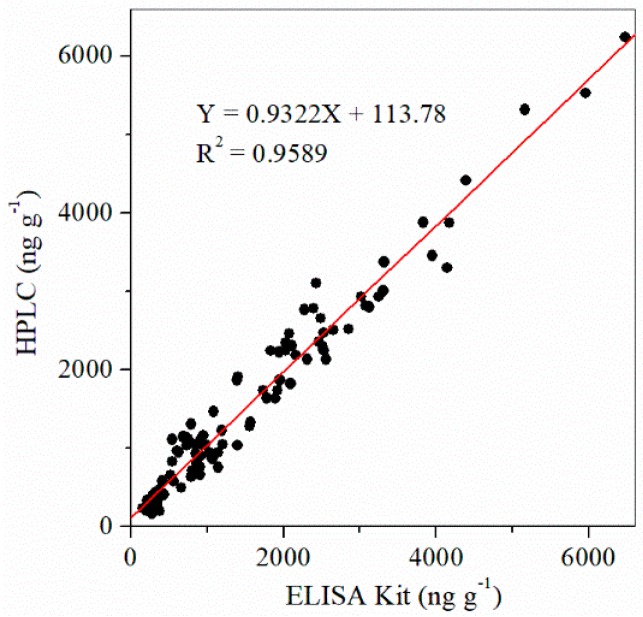
Correlation of the results of the ELISA kit and HPLC for DON (*n* = 3). HPLC, high-performance liquid chromatography.

The ELISA kit produced results largely consistent with the standardized HPLC method, and the results are presented in Figure 2. A good correlation of the results of HPLC (*Y*) and ELISA kit (*X*) was obtained (*Y* = 0.9322*X* + 113.78, *R*^2^ = 0.9589), which further indicated that the results of the ELISA kit were reliable.

## 3. Conclusions

In summary, a self-assembly ELISA kit was successfully applied to a survey of 328 agricultural product samples highly suspected of DON contamination from flour companies, feed companies, and livestock farms throughout the Chinese market. The proposed ELISA kit demonstrated sensitivity, specificity, accuracy, and the ability to be applied to large-scale screening to survey for DON contamination. Among the 328 samples, the DON detection rate was 88.7% with concentrations ranging from 200.9 to 6480.6 ng g^−1^. The highest DON concentrations were found in the DDGS samples with an average of 3204.5 ng g^−1^, and the corn meal samples had the lowest average DON level of 279.2 ng g^−1^ and the lowest DON detection rate. The wheat bran and wheat samples were the most commonly contaminated samples. This survey suggested that the DON contamination in the Chinese market is a serious issue. Our findings could provide valuable information for farmers and the government to implement the necessary measures to ensure the safety of agricultural products and human health.

## 4. Materials and Methods

### 4.1. Chemicals and Materials

The standards of DON, 3-acetyldeoxynivalenol (3-AC-DON), 15-acetyldeoxynivalenol (15-AC-DON), deoxynivalenol 3-glucoside (DON-3-G), T-2 toxin (T-2), ochratoxins A (OTA), zearalenone (ZEN), aflatoxin B_1_ (AFB_1_), and goat anti-mouse horseradish-peroxidase-conjugated antibody (GAM-HRP) were provided by Sigma Chemical Co. (St. Louis, MO, USA). Bovine serum albumin (BSA), ovalbumin (OVA), H_2_O_2_, polyoxyethylene sorbitan monolaurate (Tween-20), 3′,5,5′-Tetramethyl benzidine (TMB), sodium azide (NaN_3_), and other chemicals were provided by Aladdin Reagent Co. Ltd. (Shanghai, China).

Phosphate-buffered saline (PBS, 10 mmol L^−1^, pH 7.4), PBS containing 0.05% Tween-20 (PBST), and carbonate-buffered saline (CBS, 50 mmol L^−1^, pH 9.6) were prepared by our laboratory. The color substrate buffer contained 0.4 mmol L^−1^ TMB and 3 mmol L^−1^ H_2_O_2_ in citrate buffer (pH 5.0).

The absorbance was performed on an Infinite M1000 Pro microtiter plate reader (Tecan Group AG, Zürich, Switzerland). The washing step was performed on a Detie HBS-4009 Washer (Nanjing Detie Experimental Equipment Co. Ltd., Nanjing, China). The ELISA procedure was carried out on Jet Biofil 96-well transparent microtiter plates (Suzhou Kechuang Biotechnology Co. Ltd., Suzhou, China). The centrifugation was carried out using a Neofuge 18R Centrifuge (Heal Force Development Ltd., Hong Kong, China). The purified water was prepared using a Milli-Q purification system (Millipore Corporation, Bedford, MA, USA). The reliability of the ELISA kit was confirmed using an Aglient 1260 HPLC equipped with a diode array detector (DAD) (Agilent Technologies, Wilmington, DC, USA).

### 4.2. Preparation of Antigen and McAb

As reported, the chemical synthesis of the DON hapten was performed via the butyric anhydride derivative method [24]. Then, the DON hapten was coupled with BSA and OVA using the activated ester method to produce an immunogen antigen (DON-BSA) and coating antigen (DON-OVA), respectively [41]. The classic hybridoma technology was applied to prepare the anti-DON monoclonal antibody (McAb) [42]. After obtaining monoclonal hybridoma cells for DON, the anti-DON McAb was produced by ascite growth and purified by saturated ammonium sulfate precipitation, and it was then stored at −20 °C until use.

### 4.3. Development of the DON ELISA Kit

#### 4.3.1. ELISA Kit Procedure

(1) Coating step. The microtiter plates were coated with DON-OVA (100 μL well^−1^, in CBS) overnight at 4 °C. The plates were washed five times with washing buffer.

(2) Blocking step. The plates were blocked (200 μL well^−1^, 1% OVA in PBS) for 30 min at 37 °C and washed.

(3) Immune reaction step. The sample or standard in working buffer (50 μL well^−1^) was added, followed by the addition of the diluted anti-DON McAb (25 μL well^−1^, in PBST) and the diluted GAM-HRP (25 μL well^−1^, in PBST) into each well. Then, the plates were shaken for 10 s and incubated for 30 min at 37 °C.

(4) Coloration step. After being washed again, the color substrate buffer was added and incubated for 10 min at 37 °C.

(5) Stop step. The reaction was stopped by adding stop buffer (50 μL well^−1^).

(6) Measurement step. The absorbance was measured at the wavelength of 450 nm.

#### 4.3.2. Standard Curve

A series of concentrations of DON standards were prepared in working buffer. Triplicate determinations were carried out. The mean values of B/B_0_, where B is the absorbance value with DON and B_0_ is the absorbance value in the absence of DON, were plotted against the logarithm of the concentrations of DON standards to obtain the sigmoidal curve using the Origin Program 7.0 software (OriginLab Co., Northampton, MA, USA). The LOD and IC_50_ were obtained from a four-parameter logistic equation.

#### 4.3.3. ELISA Kit Parameters

The key parameters of the ELISA kit (concentrations of DON-OVA, anti-DON McAb, and GAM-HRP; content of methanol; concentration of Na^+^; and pH value) were studied to improve the sensitivity of the ELISA kit. The highest ratio of B_0_/IC_50_ and the lowest IC_50_ were used as the most desirable criteria for the ELISA kit. Other key buffers, compositions, and parameters were adjusted and optimized.

#### 4.3.4. CRs Experiment

The CRs of the ELISA kit toward other related mycotoxins were used to evaluate the specificity of the ELISA kit.

### 4.4. Adjustment of ELISA Kit Conditions

The optimum conditions of the ELISA kit were very important for an excellent testing technique. The components and parameters of the ELISA kit are listed in Table 5. The ELISA kit has eight components, including preprocessed plates, DON standard, antibody solution, GAM-HRP solution, color substrate buffer, stop buffer, 10 × washing buffer, and 1 × working buffer.

**Figure 3:**
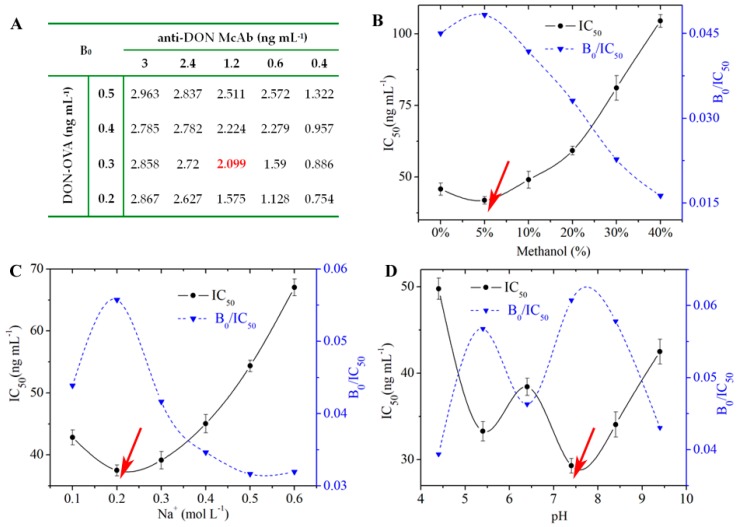
Optimization of the experimental parameters of the ELISA kit. (**A**) Optimization of the concentrations of DON-OVA and anti-DON McAb; (**B**) methanol content; (**C**) concentration of Na^+^; (**D**) pH value.

The 96-well transparent microplates were preprocessed using a coating step and a blocking step. For the key parameters, 0.3 ng mL^−1^ DON-OVA and 1.2 ng mL^−1^ anti-DON McAb were selected as the optimal dilution concentrations, when the value of B_0_ reached 2.0 (Figure 3A). The optimal dilution concentration of GAM-HRP was 25 ng mL^−1^. The working buffer, which could greatly affect the sensitivity of the ELISA kit, was adjusted. Finally, 5% methanol (Figure 3B), 0.2 mol L^−1^ Na^+^ (Figure 3C), and pH 7.4 (Figure 3D) in the working buffer were selected as the optimal working conditions of the ELISA kit for the determination of DON. For the washing buffer, a 10-fold concentration of 0.01 mol L^−1^ PBS containing Tween-20 was prepared and diluted before use. The color substrate was TMB and was oxidized by H_2_O_2_ in citrate buffer, and the stop substrate was sulfuric acid.

### 4.5. Instructions for the ELISA Kit

**Figure 4:**
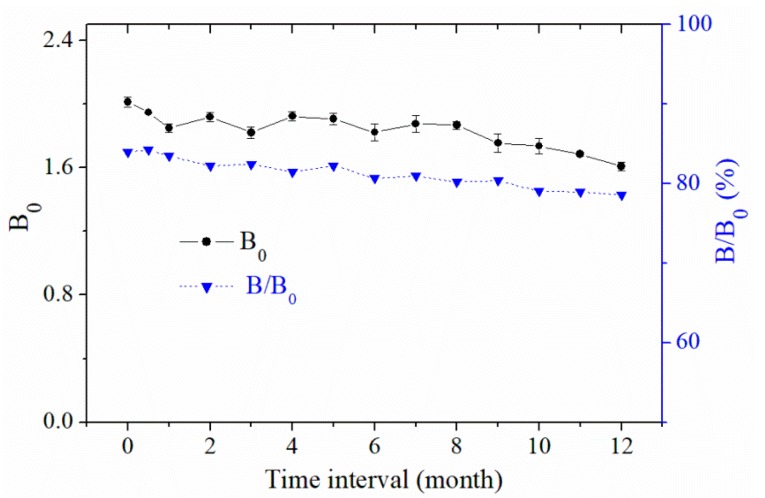
Stability experiments for the ELISA kit’s storage life. B_0_, the value of absorbance in the absence of DON; B/B_0_ (%), the ratio value of absorbance with 5 ng mL^−1^ DON and in the absence of DON.

As a biochemical test product, the ELISA kit has some special instructions for its use and storage. Firstly, the ELISA kit must be stored at 2–8 °C, the effective service life is 12 months, and the kit must not be frozen. Secondly, the microplates and reagents must be placed at room temperature (20–25 °C) for 30 min to ensure better activity and sufficient mixing before use. Microplates cannot be allowed to dry during the testing process. Stability experiments for the ELISA kit were performed and are shown in Figure 4. During storage, the values of B_0_ and B/B_0_ (%) showed an acceptable reduction. This indicated that the effective service life of the ELISA kit could be at least 12 months.

### 4.6. Spiked Samples Analysis

The free DON agricultural product samples, including wheat flour, DDGS, feed, corn, wheat bran, wheat, and corn meal samples, were selected for the recovery and matrix effect studies. These agricultural product samples were finely ground. Then, 5 g of each agricultural product sample was spiked with DON at 200, 500, and 1000 ng g^−1^ and stored for 2 h at room temperature. Next, the spiked samples were added to 10 mL of working buffer containing 20% methanol, then extracted for 10 min under ultrasonic conditions and centrifuged for 10 min at 4000 rpm. The extracted solutions were filtered and diluted using the working buffer prior to ELISA kit analysis. Each analysis was performed three times. The results of the recoveries and RSDs were used to evaluate the accuracy and precision of the ELISA kit, respectively.

To analyze the matrix effects of the agricultural product samples on the sensitivity of the ELISA kit, serial dilutions of the solutions of extracted samples were obtained in the working buffer and then analyzed using the ELISA kit. The multiple dilutions of extracted solutions resulted in a curve that was close to the DON standard curve, indicating that matrix interference was negligible; thus, they could be applied for subsequent ELISA kit determination of samples.

### 4.7. Determination in Authentic Contaminated Samples

A total of 328 agricultural product samples which were highly suspected to be contaminated with DON were collected from flour companies, feed companies, and livestock farms throughout China. According to the ELISA kit method detailed above, various agricultural product samples, such as wheat flour, DDGS, feed, corn, wheat bran, wheat, and corn meal, were prepared and analyzed.

### 4.8. Confirmation of the ELISA Kit with HPLC

To confirm the effectiveness of the proposed ELISA kit, the authentic agricultural product samples were tested using the ELISA kit and HPLC. For HPLC, the samples were tested according to the method of the national standard of China [43]. Each sample was added to 20 mL of acetonitrile-H_2_O (84:16, v/v) and extracted by sonication for 20 min and centrifugation for 5 min at 10,000 rpm. After that, the supernatants were purified through DON immuno-affinity columns (PriboLab, Beijing). The extracted phases were collected and analyzed by HPLC. The analysis was conducted using an Aglient 1260 HPLC equipped with a DAD detector. Separation was performed on an Eclipse XDB2-C18 column (250 mm × 4.6 mm × 5 μm) at 35 °C. The mobile phase was a mixture of methanol and H_2_O (20:80, v/v) at a flow rate of 0.8 mL min^−1^. The injection volume was 50 μL, and the detection wavelength was set to 218 nm. Then, the correlation of the results of the ELISA kit and those obtained from HPLC was analyzed.

## Figures and Tables

**Table 1:** Comparison of the properties of referenced and proposed immunoassays for DON.

Methods	Linear Range (ng mL^−1^)	LOD (ng mL^−1^)	IC_50_ (ng mL^−1^)	References
ELISA	10–100,000	20	3407.7	[2]
ELISA	5–1000	5.0	–	[21]
ELISA	–	0.2	18	[22]
ELISA	–	6.1	61.1	[35]
ELISA	–	–	23.4	[37]
CLEIA	1.7–170.0	0.49	17	[24]
FPIA	447.5–3780	242.0	1300	[25]
TRFIA	0.01–100	0.01	4.84	[26]
TRFIA	0.0194–100	0.0194	–	[27]
GICA	–	–	50	[28]
SPR-immunoassay	130–10,000	2.5	720	[30]
Silver staining GICA	–	–	40	[31]
Nanobody-based ELISA	2.2–62.2	1.2	8.8	[32]
Optical immunosensor	2.5–125	2.5	24	[33]
Electrochemiluminescence immunosensor	0.0001–20	0.00003	–	[34]
ELISA kit	4.9–128.9	4.9	25.2	This study

CLEIA, chemiluminescence enzyme immunoassay; FPIA, fluorescence polarization immunoassay; TRFIA, time-resolved fluoroimmunoassay; GICA, gold immunochromatographic assay; SPR, surface plasmon resonance.

**Table 2:** Cross-reactivity of the DON ELISA kit toward related mycotoxins.

Compound	IC_50_ (ng mL^−1^)	CR (%)
DON	25.2	100
3-AC-DON	442.1	5.7
15-AC-DON	>5000	<0.5
DON-3-G	>10,000	<0.3
T-2	>10,000	<0.3
OTA	>10,000	<0.3
ZEN	>10,000	<0.3
AFB_1_	>10,000	<0.3

DON, deoxynivalenol; 3-AC-DON, 3-acetyldeoxynivalenol; 15-AC-DON, 15-acetyldeoxynivalenol; DON-3-G, deoxynivalenol 3-glucoside; T-2, T-2 toxin; OTA, ochratoxins A; ZEN, zearalenone; AFB_1_, aflatoxin B_1_; CR (%), cross-reactivity = (IC_50_ of DON/IC_50_ of related mycotoxin) × 100.

**Table 3:** Accuracy and precision of DON in classified samples by the ELISA kit (*n* = 3, 40-fold dilution).

Sample	Spiked (ng g^−1^)	Mean Recovery ± SD (%)	RSD (%)	Sample	Spiked (ng g^−1^)	Mean Recovery ± SD (%)	RSD (%)
Wheat flour	200	87.1 ± 6.9	7.9	Corn	200	88.1 ± 5.7	6.5
	500	95.0 ± 5.3	5.6		500	98.4 ± 7.3	7.4
	1000	113.2 ± 9.3	8.2		1000	95.2 ± 6.2	6.5
DDGS	200	79.4 ± 7.5	9.4	Wheat	200	107.0 ± 7.6	7.1
	500	89.2 ± 6.2	7.0		500	106.7 ± 13.5	12.6
	1000	104.4 ± 5.8	5.5		1000	96.4 ± 6.3	6.5
Feed	200	77.1 ± 9.2	11.9	Wheat bran	200	76.3 ± 10.1	13.2
	500	81.7 ± 5.6	6.8		500	95.0 ± 4.4	4.6
	1000	89.4 ± 3.5	3.9		1000	96.5 ± 4.1	4.2
Corn meal	200	114.5 ± 7.8	6.8				
	500	93.3 ± 3.9	4.2				
	1000	103.7 ± 4.6	4.4				

SD, standard deviation; RSD, relative standard deviation; DDGS, distillers’ dried grains with solubles.

**Table 4:** Detection of DON contamination distribution by the ELISA kit.

Sample	Number	Average (ng g^−1^)	Range (ng g^−1^)	Detection Rate (%)	^a^ Less than 200 ng g^−1^ (%)	200–1000 ng g^−1^ (%)	1000–3000 ng g^−1^ (%)	Above 3000 ng g^−1^ (%)
Wheat flour	135	1482.4	203.3–5164.7	91.8	8.2	28.9	40.0	22.9
DDGS	17	3204.5	1201.3–6480.6	94.1	5.9	0	47.0	47.0
Feed	56	755.1	214.9–3449.3	83.9	16.1	62.5	17.9	3.5
Corn	47	445.3	200.9–2274.1	87.2	12.8	76.6	10.6	0
Wheat bran	15	1849.86	616.0–3123.4	100	0	26.7	66.7	6.6
Wheat	31	1338.1	204.8–5960.0	100	0	48.4	48.4	3.2
Corn meal	27	279.2	226.3–899.5	63.0	37	63.0	0	0

^a^ Less than 200 ng g^−1^ was not detected by the ELISA kit.

**Table 5:** Components and parameters of the ELISA kit.

Number	Composition	Parameters
1	Preprocessed plates	96-well transparent microplates (coated with 0.3 ng mL^−1^ DON-OVA and blocked with 1% OVA)
2	DON standard	5000 ng mL^−1^ in methanol (Dilute to serial standard solution using working buffer before use)
3	Antibody solution	6 mL (1.2 ng mL^−1^ anti-DON McAb in PBS with 2% BSA and 0.05% NaN_3_)
4	GAM-HRP solution	6mL (25 ng mL^−1^ GAM-HRP in PBS with 2% BSA and 0.05% NaN_3_)
5	Color substrate buffer	15 mL (0.4 mmol L^−1^ TMB and 3 mmol L^−1^ H_2_O_2_ in citrate buffer, pH 5.0)
6	Stop buffer	7 mL (2 mol L^−1^ H_2_SO_4_ in H_2_O)
7	10 × Washing buffer	40 mL (10 × PBST, pH 7.4)
8	1 × Working buffer	50 mL (5% methanol, 0.2 mol L^−1^ Na^+^, pH 7.4 in PBS)

OVA, ovalbumin; McAb, monoclonal antibody; GAM-HRP, goat anti-mouse horseradish-peroxidase-conjugated antibody; TMB, 3′,5,5′-Tetramethyl benzidine; BSA, bovine serum albumin; PBS, phosphate-buffered saline.
